# ERAD and how viruses exploit it

**DOI:** 10.3389/fmicb.2014.00330

**Published:** 2014-07-03

**Authors:** Hyewon Byun, Yongqiang Gou, Adam Zook, Mary M. Lozano, Jaquelin P. Dudley

**Affiliations:** Department of Molecular Biosciences, Center for Infectious Diseases and Institute for Cellular and Molecular Biology, The University of Texas at AustinAustin, TX, USA

**Keywords:** ERAD, immune response, retrotranslocation, ubiquitination, proteasomal degradation, retrovirus, herpesvirus, polyomavirus

## Abstract

Endoplasmic reticulum (ER)-associated degradation (ERAD) is a universally important process among eukaryotic cells. ERAD is necessary to preserve cell integrity since the accumulation of defective proteins results in diseases associated with neurological dysfunction, cancer, and infections. This process involves recognition of misfolded or misassembled proteins that have been translated in association with ER membranes. Recognition of ERAD substrates leads to their extraction through the ER membrane (retrotranslocation or dislocation), ubiquitination, and destruction by cytosolic proteasomes. This review focuses on ERAD and its components as well as how viruses use this process to promote their replication and to avoid the immune response.

## INTRODUCTION

Although endoplasmic reticulum (ER)-associated degradation (ERAD) has been most thoroughly defined in yeast, recent studies in higher organisms have revealed the conservation of this process and its components. Multiple diseases, including Parkinson’s, Alzheimer’s, cancer, and infectious processes, result from failure of ERAD, confirming its significance for correct cell function. Predictably, viruses have exploited various aspects of this key cellular machinery to further their propagation. Nonetheless, the complexity of ERAD and the number of players involved necessitates a review of its features prior to a description of how viruses have manipulated ERAD to their advantage. In understanding how viruses exploit ERAD, we learn more about the cellular process, but also how we might alter the outcome of viral diseases.

## THE ERAD PROCESS

A majority of newly synthesized proteins in mammalian cells are either misfolded or misassembled (Hoseki et al., 2010). Approximately 30% of new proteins are synthesized in association with the ER (Brodsky and Wojcikiewicz, 2009). The ER quality control system both senses and disposes of terminally misfolded proteins by ERAD, a process that is conserved in eukaryotes (Vembar and Brodsky, 2008; Merulla et al., 2013). This process detects misfolded proteins in the ER lumen, and then extracts them through membrane channels in an energy-dependent manner for delivery to cytosolic proteasomes (Olzmann et al., 2013). Protein extraction through ER membrane channels is known as dislocation or retrotranslocation (Hampton and Sommer, 2012). Because protein folding depends on multiple cellular components (Merulla et al., 2013), protein overexpression or the presence of mutant proteins may sequester limiting components, leading to accumulation of misfolded proteins in the ER lumen. A more general failure of the ERAD process may occur if proteins are unable to fold within a reasonable time, resulting in inefficient retrotranslocation and proteasomal degradation. Levels of ERAD-associated factors also may be affected by the intraluminal concentration of misfolded proteins. Inability of the ERAD system to destroy misfolded proteins is associated with more than 60 diseases, including neurological illnesses (Alzheimer’s and Parkinson’s), cystic fibrosis, infectious diseases, diabetes, and cancer (Guerriero and Brodsky, 2012). Particularly relevant to the subject of this review, viruses can produce large quantities of glycoproteins in a short period of time, which may overwhelm ERAD, leading to the accumulation of misfolded proteins, cell death, and associated pathology (Franz et al., 2014).

Although ERAD is vital to the maintenance of healthy cells, many parts of this process are not well characterized. Multiple aspects of ERAD have been described in yeast (Thibault and Ng, 2012), including the nature of the ER channel and the components needed to identify misfolded proteins during and after translation. Protein translocation across the ER membrane is the prerequisite for ERAD. Translation of many transmembrane proteins involves recognition of a hydrophobic signal peptide (SP) emerging from the ribosome by signal recognition particle (SRP), which is associated with the trimeric Sec61 complex. Many of the SPs are cleaved by signal peptidase, which is associated with the luminal side of the translocon (Auclair et al., 2012). The Sec61 complex provides the aqueous channel for co-translational transfer of proteins across the ER membrane (Loibl et al., 2014).

**FIGURE 1:**
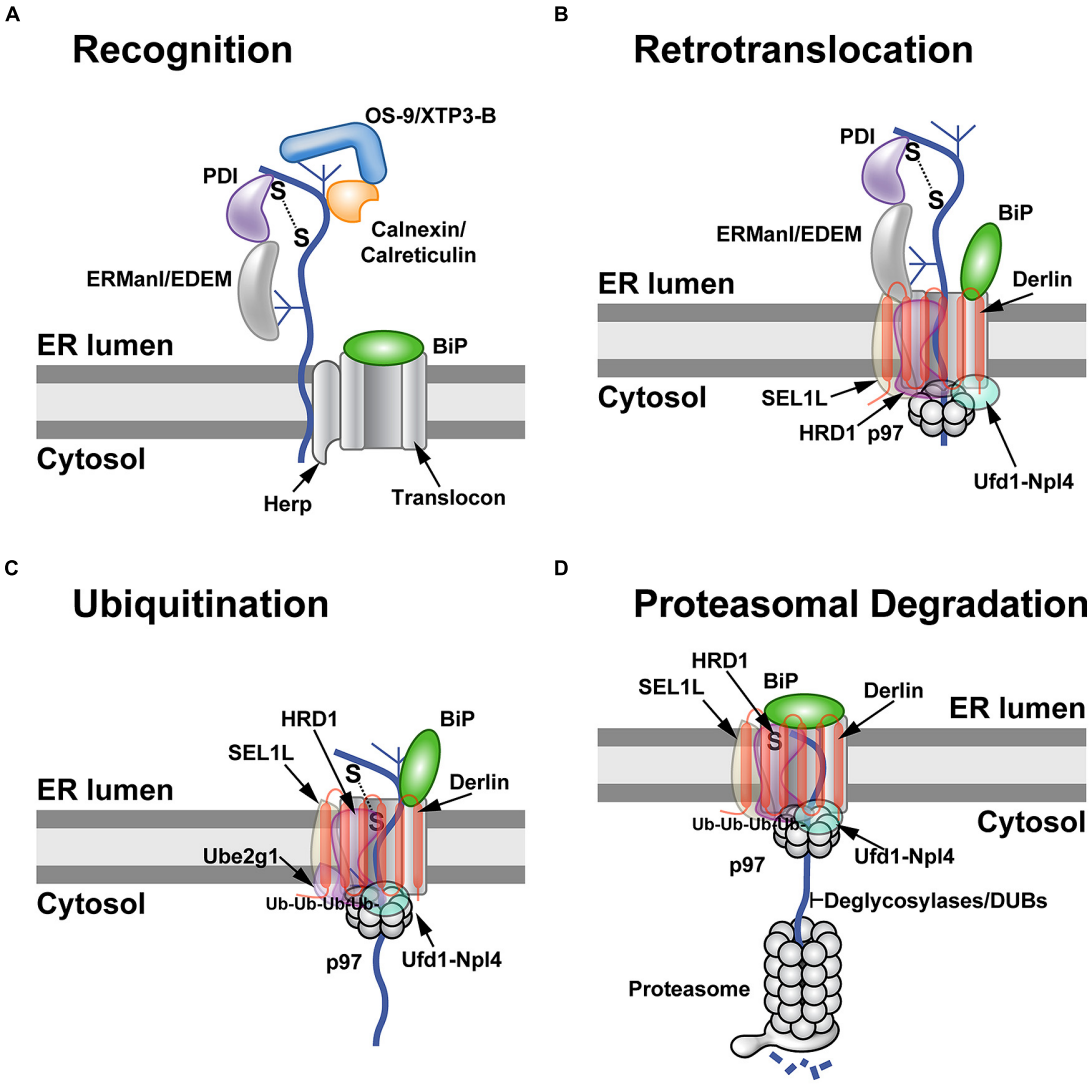
**The ERAD process. (A)** Substrate recognition. Many nascent polypeptides (curved line) have one or more high-mannose carbohydrates (shown as a branched structure), which must be recognized and processed in a timely manner to allow exit from the ER. Binding of these ER-luminal proteins to substrates is affected by folding to their native conformations. Folding involves formation and breakage of disulfide bonds by members of the PDI family, such as ERp57 and ERp72, and is facilitated by chaperone proteins, such as BiP. Specific carbohydrates are bound by different chaperones/lectins in the ER lumen. These proteins include ERManI, EDEM, OS-9, XTP3-B, calreticulin, and calnexin. Recognition of ERAD substrates probably results in assembly of the retrotranslocon (shown here as Herp and the translocon/BiP complex). Herp is thought to facilitate oligomerization of the Hrd1 E3 ligase. BiP binds to a number of glycosylated and non-glycosylated ERAD substrates and provides a barrier on the ER luminal side of the translocon. **(B)** Retrotranslocation. Recognition of misfolded or misassembled proteins triggers the assembly of the retrotranslocon. Current evidence indicates that multiple types of retrotranslocons are possible (see text). A typical retrotranslocon/dislocon is shown containing Derlin, the E3 ligase Hrd1 and its partner Sel1L, which then recruits the cytosolic ATPase p97. Derlin has 6 transmembrane domains with both the N-terminus and C-terminus in the cytosol. Presumably some or all of the recognition components, such as PDI and ERManI, disengage as the substrate passes through the translocon. All retrotranslocation events appear to involve p97. The retrotranslocon is shown with BiP opening the Sec61 channel for substrate passage into the cytosol. **(C)** Ubiquitination of ERAD substrates. Retrotranslocation exposes ERAD substrates to cytosolic E1 (unknown), E2 (shown here as Ube2g1), and E3 enzymes (e.g., Hrd1). A polyubiquitin chain is produced as the substrate is engaged by the E2 and E3 proteins. Multiple E3s may be responsible for the polyubiquitin chains that then bind to the p97 partner proteins, Ufd1 and Npl4. The substrate is shown moving through the translocon into the center of the p97 hexamer. **(D)** Proteasomal degradation. Once the substrate has been retrotranslocated, the BiP protein seals the luminal side of the translocon. The retrotranslocon may then be disassembled prior to engagement of a new substrate. The retrotranslocated proteins must be modified by removal of carbohydrate and ubiquitin chains for insertion into the narrow channel of the proteasome. It is possible that p97 substitutes for the 19S lid, which provides access to the proteasome channel and the energy for unfolding of substrates. Degraded polypeptides are shown emerging from the 19S lid. This model suggests that there are retrotranslocon-specific proteasomes.

Recent evidence indicates that translocation across the ER membrane can occur through an SRP-independent process (Denic, 2012; Johnson et al., 2013). Based on recent experiments in yeast, more than 40% of signal-containing proteins fail to use SRP, including tail-anchored (TA) proteins and short secretory proteins (Johnson et al., 2012; Ast et al., 2013). Instead, these proteins are targeted by the GET pathway to the Sec61 translocon that is associated with the Sec 62/63 complex rather than through docking to the SRP receptor (Rapoport, 2007; Ast et al., 2013). One large class of SRP-independent proteins includes the glycosylphosphatidylinositol (GPI)-anchored proteins, which contain both an N-terminal signal sequence and a C-terminal GPI anchor (Ast et al., 2013). This N-terminal signal is less hydrophobic than typical SRP targets. Furthermore, the Sec61 translocon has been implicated as the channel for retrotranslocation (Kiser et al., 2001), and it has been proposed that protein transfer can be either forward or reverse with respect to the ER lumen (Johnson and Haigh, 2000). Therefore, Sec61 appears to complex with a number of different proteins, leading to a highly flexible and dynamic structure, where association with different proteins/protein complexes leads to transit in or out of the ER (**Figure 1**).

## SUBSTRATE RECOGNITION FOR ERAD

Reports in yeast indicate that proteins can be O-mannosylated prior to N-glycosylation (Ecker et al., 2003), and both types of glycosylation are believed to occur co-translationally (Loibl et al., 2014). These glycosylases also have been shown to be associated with the translocon (Chavan and Lennarz, 2006), and experiments indicate competition for different glycosylation sites (Loibl et al., 2014). The protein *O*-mannosyl transferases (PMTs) and the oligosaccharyltransferases (OSTs) are transmembrane proteins, but the latter catalyzes addition of oligosaccharides to nascent polypeptides on asparagine residues (Breitling and Aebi, 2013). The OSTs prefer NxT/S sequences in an unfolded or flexible protein domain, and the unfolded state may be facilitated by the OST complex associated with the translocon (Breitling and Aebi, 2013). Glycosylation near the C-terminal end of the protein is less efficient, perhaps due to competition between OSTs and protein folding (Ben-Dor et al., 2004; Breitling and Aebi, 2013). PMTs also are essential for ERAD in yeast. A Δ*pmt* mutant showed increased degradation of a typical ERAD substrate (Arroyo et al., 2011). Moreover, addition of oligosaccharides can be prevented by nearby cysteines and disulfide bond formation (Allen et al., 1995). Thus, glycosylation is one determinant of the correct folding of a protein in the ER lumen (Breitling and Aebi, 2013; **Figure 1A**).

The oligosaccharides on ER luminal proteins are critical for their correct folding or selection for ERAD. The nascent N-glycosylated protein has a three-branch structure with glucose_3_-mannose_9_-*N*-acetylglucosamine_2_-asparagine (Aebi et al., 2010; Merulla et al., 2013). Trimming of the first two glucose residues on one branch then allows interactions with two ER-resident chaperone/lectin proteins, calnexin and calreticulin, which may lead to protein folding (Brodsky, 2012). Removal of the third glucose causes release from these lectins and exit from the ER (Smith et al., 2011; Olzmann et al., 2013), but re-addition of this glucose by UDP-glucose:glycoprotein glucosyltransferase allows reassociation (Shenkman et al., 2013). Proteins retry folding until removal of three or four mannose residues triggers ERAD (Lederkremer and Glickman, 2005; Shenkman et al., 2013). Correctly folded proteins leave the ER after one or two mannose residues have been cleaved (Shenkman et al., 2013). Mannose removal is achieved using ER mannosidase I (ERmanI), the ER degradation-enhancing α-mannosidase-like proteins (EDEMs) and/or the Golgi-resident protein Man1C1 (Gonzalez et al., 1999; Hirao et al., 2006; Olivari et al., 2006; Hosokawa et al., 2007). Several lectins, OS-9 and XTP3-B, then interact via their MRH domains with the mannose-trimmed proteins, allowing their association with the retrotranslocon (Bernasconi et al., 2008; Christianson et al., 2008; Hosokawa et al., 2008). OS-9 and XTP3-B also associate with different proteases, LONP2 and carboxypeptidase vitellogenic-like protein (CPVL), respectively, suggesting that some substrates may be partially degraded prior to dislocation (Christianson et al., 2012; Olzmann et al., 2013). Nonetheless, multiple attempts are made to refold proteins before their triage through ERAD. The role of chaperones includes recognition of inappropriate glycosylation as well as refolding efforts, but proteins delivered to the retrotranslocon may require unfolding and partial proteolysis to allow their transit through the narrow membrane channel (Gogala et al., 2014).

Non-glycosylated proteins can be subjected to ERAD, but detection of misfolding of these proteins does not involve calnexin and calreticulin (Brodsky, 2012). Notably, the non-lectin chaperone BiP is involved in ERAD targeting of both types of proteins (Ushioda et al., 2013), yet also serves to prevent leakage of calcium out of the ER lumen (Schäuble et al., 2012). In addition, targeting of unglycosylated proteins to the proteasomes involves EDEM1 (Shenkman et al., 2013), which, like BiP, recognizes misfolded glycoproteins, as well as the transmembrane Herp protein (Usa1p in yeast; Okuda-Shimizu and Hendershot, 2007). Both glycosylated and their non-glycosylated derivatives are recruited to the ER-derived quality control compartment (ERQC) near the nucleus in the presence of a proteasomal inhibitor (Shenkman et al., 2013). Thus, these studies suggest that targeting of misfolded proteins for ERAD is similar for glycoproteins and non-glycosylated proteins (Shenkman et al., 2013).

Interaction of lectin-type and other chaperones with ERAD substrates allows association with members of the protein disulfide isomerase (PDI) family, which generally are characterized by one or more thioredoxin-like motifs (CXXC; Brodsky and Skach, 2011). Interestingly, these proteins can form, break, or rearrange disulfide bonds as well as act as chaperones (Benham, 2012). The yeast PDI family is composed of five members (Pdi1, Mpd1, Mpd2, Eug1, and Eps1), although only Pdi1 is essential (Farquhar et al., 1991). In mammalian cells, PDI is one of the best characterized family members, but there are at least 21 such enzymes (Benham, 2012; Grubb et al., 2012). PDI family proteins are generally confined by a KDEL retention sequence (Benham, 2012) to the ER, which has an oxidizing environment (Costantini et al., 2013). The oxidoreductase ERp57, which is localized near the ER-Golgi intermediate compartment (ERGIC), may provide some protection for proteins that might be routed for ERAD by calnexin (Frenkel et al., 2004). In addition, some PDI members can escape the secretory system and appear at the cell surface (Benham, 2012). For example, a disintegrin and metalloproteinase (ADAM17; also known as tumor necrosis factor alpha-converting enzyme or TACE) has been shown to be regulated by an extracellular activity of PDI (Bass and Edwards, 2010; Willems et al., 2010; Düsterhöft et al., 2013). PDI members also have a role in ERAD, with different requirements for different substrates (Grubb et al., 2012). In hepatic cells, PDI promotes the folding of apolipoprotein B (ApoB) through its chaperone activity, whereas ERp57 or ERp72 expression leads to ERAD (Grubb et al., 2012). Further, various cell types express different PDI proteins, allowing differential regulation of substrates (Benham, 2012; Pescatore et al., 2012) and, presumably, their ERAD targeting.

Protein folding involves both formation of disulfide bonds and cis/trans isomerization of peptide bonds preceding proline residues (Hebert and Molinari, 2007). Certain ERAD substrates appear to be dependent on proline isomerization (Bernasconi et al., 2010b), and such refolding events may be necessary for transit through the retranslocon by elimination of turns in substrate secondary structure (Määttänen et al., 2010). ERAD requirements for peptidyl-prolyl *cis/trans* isomerases (PPIs) depend on whether the substrate is strictly in the ER lumen or is tethered to the ER membrane (Bernasconi et al., 2010b). The PPI protein cyclophilin B was needed for ERAD of a luminal target, but not the same target with a transmembrane domain (Bernasconi et al., 2010b). Requirement for PPIs during ERAD may depend on proline residues in the *cis* configuration (Bernasconi et al., 2010b), potentially by conversion into *trans* peptidyl–prolyl bonds, thus eliminating secondary structures that hinder retrotranslocation (Määttänen et al., 2010).

## RETROTRANSLOCATION

Mammalian cells have ERAD factors that are not present in yeast. As observed for other pathways (Tsai and Weissman, 2012), ERAD components identified in yeast have multiple family members in higher eukaryotes; e.g., instead of a single Derlin in yeast (Der1p), mammalian cells have three proteins (Derlin-1, -2, and -3; Oda et al., 2006). Derlins are multiple membrane-spanning domain proteins that have been proposed to be part of the retrotranslocon channel (Ye et al., 2005) and/or regulatory factors for retrotranslocation (Brodsky, 2012; **Figure 1B**). In addition, Derlin-3 has a cell-type specific distribution (Oda et al., 2006), suggesting that recognition of certain substrates may be involved in its function. Derlins are related to rhomboid proteases, such as RHBDL4, which is an ER-resident transmembrane protein that cleaves unstable single-membrane-spanning or polytopic membrane proteins (Fleig et al., 2012). RHBDL4 also is upregulated by ER stress and binds to the cytosolic AAA ATPase p97 (see below; Fleig et al., 2012). In contrast to the rhomboid proteases, the Derlins lack proteolytic activity, suggesting that these proteins bind to ERAD substrates and target them to E3 ligases for ubiquitination and to p97 for membrane extraction (Brodsky, 2012). Cleavage of ERAD substrates by RHBDL4 (Fleig et al., 2012), SP peptidase (SPP; Loureiro et al., 2006), or proteases associated with OS-9 and XTP3-B (Olzmann et al., 2013) may occur prior to retrotranslocation of some substrates (Tsai and Weissman, 2012). On the other hand, it has been proposed that Derlins form a six-transmembrane structure with a gate that allows association and unfolding of substrates or access to other retrotranslocon components, such as p97 (see below; Olzmann et al., 2013). The p97 ATPase (Cdc48 in yeast) is bound to Derlin-1 and Derlin-2 through their SHP domains (Greenblatt et al., 2011).

Suppressor/enhancer of Lin12-like (SEL1L) appears to link luminal factors that recognize misfolding and inappropriate glycosylation, such as OS-9, XTP3-B, EDEMs, ERdj5, and the PDI protein ERp90, to components of the retrotranslocon (Olzmann et al., 2013; Williams et al., 2013). The transmembrane SEL1L protein (Hrd3p in yeast) also participates in regulation of ERAD by sequestering EDEM1 and OS-9 into ER-derived vesicles known as EDEMosomes (Bernasconi et al., 2012a). Inducible knockout of Sel1L in mice leads to death of adult mice from acute pancreatic atrophy (Sun et al., 2014). Sel1L expression is required for stability of the E3 ligase hydroxymethylglutaryl reductase degradation protein 1 (Hrd1), and its loss leads to ER stress and attenuates translation, leading to cell death. Other proteins have been described, such as Erlins 1 and 2 and TMUB1, which may act as adapters between polytopic membrane substrates and E3 ligases (Olzmann et al., 2013).

## UBIQUITINATION

The ubiquitin ligases (E3s) have been proposed to be a structural part of the retrotranslocon channel (Brodsky, 2012), but their role is considerably more complex (**Figure 1C**). Several E3 ligases associated with ERAD are multiple membrane-spanning proteins with cytosolic RING domains (Smith et al., 2011; Ruggiano et al., 2014). In yeast, where ERAD has been studied most extensively, a prototypical transmembrane E3, such as Hrd1p (also called SYVN1; Nadav et al., 2003; Kikkert et al., 2004), can promote ERAD of a luminal substrate (ERAD-L). The ERAD process also involves Hrd3p (SEL1L in metazoans) as well as Usa1p and Der1p (Carvalho et al., 2010). Herp may assist with Hrd1 oligomerization (Carvalho et al., 2010), Nevertheless, the other components appear to be dispensable if Hrd1p is overexpressed, consistent with a role for Hrd1p in ERAD substrate transfer across the membrane (Carvalho et al., 2010), although such overexpression may be toxic due to inappropriate protein degradation (Denic et al., 2006). Thus, protein adapters appear to be necessary to achieve substrate specificity (Smith et al., 2011).

Hrd1p-mediated ERAD requires oligomerization and transmembrane domains as well as ubiquitin ligase activity (Carvalho et al., 2010). Overexpression of a dominant-negative RING mutant of the HRD1 ligase prevented ERAD of a non-glycosylated substrate, but a dominant-negative Fbs2 mutant (a component of SCF E3 ligases) did not (Shenkman et al., 2013). Dependence on HRD1 also is affected by tethering of the substrate to the ER membrane. Splice variants of the human beta-site amyloid precursor cleaving enzyme (BACE) with the same deletion mutation in the ectodomain are degraded through HRD1 if they are luminal (ERAD-L_S_ substrates), but disposal occurs in a HRD1-independent manner if the variant has a transmembrane domain (ERAD-L_M_ substrates; Bernasconi et al., 2010a). Therefore, HRD1 recognizes substrates for ubiquitination and, perhaps, modifies the translocon in the ER membrane.

Multiple E3 ligases participate in ERAD. These ligases include the transmembrane proteins gp78/AMFR (Fairbank et al., 2009), TRC8 (Stagg et al., 2009), RMA1/RNF5 (El Khouri et al., 2013), MARCH6/TEB4 (Doa10 in yeast; Kreft and Hochstrasser, 2011; Olzmann et al., 2013), and CHIP (Matsumura et al., 2013). An additional 40-50 membrane-spanning E3s may be involved in ERAD (Stagg et al., 2009). Other E3 ligases associated with ERAD are localized to the cytosol, where they recognize misfolded glycoproteins that already have been retrotranslocated (Yoshida et al., 2005; Shenkman et al., 2013). These ubiquitin ligases are members of the cytosolic SCF (S-phase kinase-associated protein 1 (Skp1)-Cullin 1 (Cul1)-F-box) family, where the F-box components of the SCF complex recognize the N-glycans of the retrotranslocated substrate, e.g., Fbs1 and Fbs2 (Yoshida, 2007). Furthermore, E3s may work together to direct substrates for degradation (Olzmann et al., 2013).

## PROTEASOMAL DEGRADATION

The p97 protein (Cdc48 in yeast) is a member of the AAA ATPase family (Erzberger and Berger, 2006) that functions during ERAD in a complex with several cofactors that have a ubiquitin-X (UBX) or UBX-like domain (Schuberth and Buchberger, 2008; **Figure 1**). These cofactors include the heterodimer nuclear protein localization homolog 4 (Npl4)–ubiquitin fusion degradation 1 (Ufd1; Meyer et al., 2012; Wolf and Stolz, 2012), p47, UBXD1, UBXD7, Ufd3/PLAA, VCIP135, and Ataxin-3 (Meyer et al., 2012). The UFD1L and NPL4 proteins are believed to form a heterodimer, where NPL4 is needed to stabilize UFD1L (Nowis et al., 2006). The heterodimer acts as a substrate adapter to the p97 ATPase associated with the retrotranslocon (Bays and Hampton, 2002). UFD1L and NPL4 bind to K48-linked and K63-linked polyubiquitin chains, respectively, which have been added by E3 ligases associated with the retrotranslocon (Ye et al., 2003; Komander et al., 2009).

In yeast, the Cdc48 ATPase binds to the Hrd1 E3 ligase in a RING-dependent manner (Hampton and Sommer, 2012), and the transmembrane Ubx2 (Sel1) protein acts as an adapter using a UBA domain (Neuber et al., 2005; Schuberth and Buchberger, 2005). Several other ubiquitin ligases bind p97 directly or through cofactors (Alexandru et al., 2008). The p97 cofactors act as ubiquitin-binding proteins, although p97 also has ubiquitin-binding activity (Ye et al., 2003; Meyer et al., 2012). The adapter-p97 complexes may recognize different substrates and perform independent functions, such as membrane protein segregation and trafficking, as well as directing substrates to the proteasome (Ritz et al., 2011). Alternatively, other models suggest that Derlins are involved in unfolding of substrates as well as providing contacts with p97 and its associated factors (Greenblatt et al., 2011). The p97 ATPase binds ubiquitin chain editors that can extend shorter chains as well as deubiquitinating enzymes (DUBs; Jentsch and Rumpf, 2007; Sowa et al., 2009). Two ATPase domains (D1 and D2; Meyer et al., 2012) within p97 form two stacked hexameric rings that provide the energy for protein remodeling and substrate extraction from the membrane or through the retrotranslocon (Hampton and Sommer, 2012). Mutations in the D2 domain result in dominant-negative proteins that bind, but fail to release, substrates (Pye et al., 2006). Mutant proteins have been widely used to study p97 function in ERAD and its myriad other activities (Meyer et al., 2012). Cytosolic chaperones, such as Hsp70, also may provide energy for extraction of membrane proteins with misfolded cytoplasmic domains (ERAD-C substrates; Taxis et al., 2003; Hrizo et al., 2007).

Once extraction from the ER membrane has occurred, p97 recruits peptide N-glycanase (PNGase) to cleave N-linked glycans from glycosylated substrates (Hirsch et al., 2003; Li et al., 2006; **Figure 1D**). In addition, p97 binds to a deubiquitinating enzyme YOD1, presumably so that polyubiquitin chains will not interfere with insertion into the proteasome (Ernst et al., 2009). The proteasome is a highly complex structure with a 19S lid that has an ATPase activity very similar to that of p97 (Lipson et al., 2008; Matouschek and Finley, 2012). These enzymes may function synergistically to deliver substrates to the 20S core (Hampton and Sommer, 2012). Alternatively, p97 may deliver certain substrates directly to the proteasome core (Matouschek and Finley, 2012). The proteasome core is composed of 28 subunits arranged into four rings, each composed of seven subunits (Bhattacharyya et al., 2014). Proteolytic activity is sequestered in the center of a narrow chamber formed by the rings and, therefore, only unfolded proteins can enter the chamber (Groll et al., 2000). The 19S lid, p97, or other activators provide docking for substrates and substrate modifying proteins as well as regulated opening of the chamber to allow access of unfolded proteins for degradation in the 26S core (Bhattacharyya et al., 2014).

Many questions remain about ERAD components and how they identify and interact with different substrates. Similar to our analysis of other cellular and molecular biological processes through virology, studies of viruses that use ERAD are likely to prove insightful.

## VIRAL MANIPULATION OF THE IMMUNE RESPONSE BY ERAD

**FIGURE 2:**
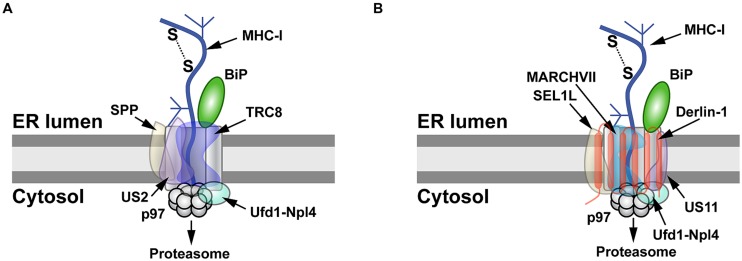
**MHC-I degradation by the herpesvirus US2 and US11 proteins. (A)** Retrotranslocation of MHC-I by US2. US2 targets MHC-I molecules for retrotranslocation by a process that requires signal peptide peptidase (SPP), the E3 ligase TRC8, and the Ufd1-Npl4-p97 complex. SPP may induce partial degradation of the substrate prior to its proteasomal entry. **(B)** Retrotranslocation of MHC-I by US11. MHC-I is retrotranslocated after US11 recruits SEL1L, Derlin-1, the E3 ligase MARCHVII, and p97. It is not clear whether either degradation of MHC-I by US2 or US11 involves the adapter complex Ufd1-Npl4, which recognizes different types of polyubiquitin chains.

The ability of viruses to cause persistent infections is a consequence of downregulation or subversion of the immune response. The herpesviruses are known to cause persistent infections. One well-studied example of herpesvirus manipulation of the immune response is reduced cell expression of major histocompatibility complex class 1 (MHC-I) molecules by the viral proteins US2 and US11 (Wiertz et al., 1996). Both proteins are transmembrane glycoproteins and bind to newly made MHC-I to initiate retrotranslocation. Despite their similar function, US2 and US11 use different pathways for MHC-I degradation (**Figure 2**). US2-mediated degradation of MHC-I is independent of Derlin-1 and involves SPP (Loureiro et al., 2006), which cleaves many SPs following their removal from nascent ER-bound pre-proteins (Voss et al., 2013). Using an siRNA screen, TRC8 was identified as the E3 ligase involved in MHC-I degradation by US2, but knockdown of this transmembrane RING-type E3 had no effect on US11-mediated destruction of MHC-I (Stagg et al., 2009). The US2 cytosolic tail interacts with SPP and the p97 ATPase (Chevalier and Johnson, 2003; Loureiro et al., 2006), whereas TRC8 and US2 bind through their transmembrane domains (Stagg et al., 2009; **Figure 2A**).

Unlike the Derlin-independent mechanism proposed for US2, studies of the US11 protein facilitated identification of Derlin-1 and SEL1L as ERAD components (**Figure 1**; Lilley and Ploegh, 2004; Ye et al., 2004; Mueller et al., 2006). US11 does not require SPP for MHC-I degradation (Loureiro et al., 2006), but appears to interact with the E3 ligase MARCHVII/axotrophin (Flierman et al., 2006). The cytosolic domain of MHC-I is required for US11-mediated ERAD targeting (Story et al., 1999; Barel et al., 2003), and deletion of the C-terminal valine of MHC-I reduced interaction with Derlin-1 (Cho et al., 2013a). The ER luminal domain also affects degradation (Barel et al., 2003). In addition, MHC-I substituted with the transmembrane domain of US11 caused interaction with Derlin-1 and proteasomal degradation (Cho et al., 2013b). The p97 ATPase does not appear to interact directly with MHC-I, but requires the interaction of MHC-I cytosolic domain with the C-terminal domain of Derlin-1 (Cho et al., 2013a). Cho et al. speculated that US11 recognizes MHC-I through its cytosolic domain and transfers it to Derlin-1, which then interacts with the p97 ATPase for membrane dislocation (Cho et al., 2013a; **Figure 2B**). Therefore, studies of the herpesvirus US2 and US11 proteins revealed that the same substrate does not always use the same ERAD pathway, and presumably these viral proteins act as adapters that recognize different parts of MHC-I for targeting to the dislocon.

Herpesviruses use another mechanism to decrease levels of MHC-I. The mouse gammaherpesvirus 68 (MHV68) encodes an E3 ligase (mK3) that ubiquitinates newly made MHC-I heavy chains for proteasomal degradation (Boname and Stevenson, 2001). The mK3 ligase also is associated with the transporter-associated with antigen processing (TAP) as well as p97 and Derlin-1 (Wang et al., 2006). Polyubiquitination of MHC-I did not require lysines (Wang et al., 2005), but could occur on serine and threonine residues in the heavy chain C-terminal tail via the recruitment of the Ube2j2 E2 enzyme (see **Figure 1**; Wang et al., 2007, 2009; Herr et al., 2009). These data indicate that multiple ERAD mechanisms can be used by viruses to diminish the adaptive immune response.

Like the herpesviruses, retroviruses also manipulate the immune system through ERAD. Early studies indicated that human immunodeficiency virus type 1 (HIV-1)-infected cells had decreased levels of both CD4 mRNA and protein (Hoxie et al., 1986). CD4 acts as the receptor for binding the viral envelope (Env) protein (McClure et al., 1987). Furthermore, CD4 participates in T-cell activation by binding to both the T-cell receptor and MHC class II molecules on antigen-presenting cells. CD4+ T cells secrete cytokines that control antibody production, phagocytic cell function, and cytotoxic T-cell responses, making them crucial for adaptive immune responses (Tubo and Jenkins, 2014). HIV-1 encodes a number of accessory proteins, including Vpu, which are not required for virus replication in tissue culture, but contribute to viral pathogenesis (Strebel, 2013). Expression of Vpu and CD4 by transient transfection showed dramatic decreases in CD4 levels, and CD4 depletion was dependent on serines 52 and 56 in Vpu (Magadán et al., 2010).

**FIGURE 3:**
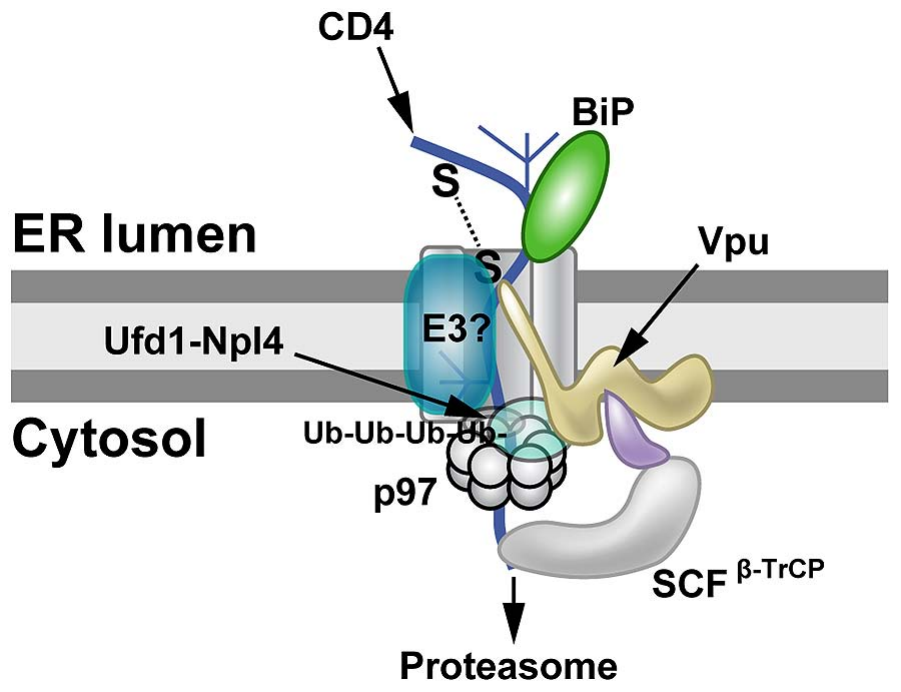
**Proteasomal targeting of CD4 by HIV-1 Vpu.** The transmembrane protein Vpu recruits the E3 ligase complex SCF^β-TrCP^. Knockdown of both β-TrCP1 and β-TrCP1 (shown to be contacting Vpu) can prevent CD4 degradation, suggesting that either F-box protein can provide a functional SCF complex for ubiquitination (Magadán et al., 2010). Another E3 ligase (E3?) also may be involved. The p97 ATPase with the adapters Ufd1 and Npl4 are required for CD4 degradation, but the UFD1L protein recognizes polyubiquitinated CD4. Lysine and serine/threonine residues in the CD4 cytosolic tail are needed for ubiquitination (Magadán et al., 2010).

Vpu-induced CD4 degradation has been shown to involve the ERAD system. Knockdown of both β-TrCP1 and β-TrCP2 largely prevented Vpu-mediated CD4 loss (Magadán et al., 2010). β-TrCP1 and β-TrCP2 (also known as FBW1A, FBXW1, FBXW1A, or FWD1) are F-box proteins containing WD40 domains, which are associated with the SCF family of ubiquitin ligases (**Figure 3**). These protein complexes are linked to regulation of multiple pathways involving cell cycle checkpoints, NFκB, and Wnt (Skaar et al., 2013). In addition, knockdown of p97, UFD1L (also called Ufd1) or NPL4 (see **Figure 1C**) blocked depletion of CD4 (Magadán et al., 2010). Mutations that prevented ATP binding or hydrolysis by p97 failed to affect CD4 levels (Magadán et al., 2010). These experiments indicated that Vpu uses ERAD to degrade CD4, but also prevents cell surface expression by retaining CD4 in the ER, probably through transmembrane domain interactions (Magadán et al., 2010). Moreover, Vpu used an atypical E3 ligase to induce ERAD (Margottin et al., 1998), and this process involved SCF^β-TrCP^ ubiquitination of the CD4 cytosolic tail on lysine, serine, and threonine residues (Magadán et al., 2010). Thus, Vpu may act as an adapter between CD4, retrotranslocon components, and a cytosolic E3 ligase. CD4 degradation promotes HIV-1 infection by preventing re-infection, facilitating virus release by avoiding Env–CD4 interactions during their trafficking to the cell surface, and minimizing adaptive immune responses (Lanzavecchia et al., 1988; Willey et al., 1992; Argañaraz et al., 2003).

HIV-1 Vpu also targets another cellular protein, tetherin/BST-2, for ERAD (Neil et al., 2008; Mangeat et al., 2009). Tetherin is an unusual type II membrane protein with an N-terminal transmembrane segment and a C-terminal GPI anchor (Kupzig et al., 2003; Sauter, 2014). Moreover, two tetherin monomers are bound together by disulfide bonds (Ishikawa et al., 1995; Kupzig et al., 2003). Using a unique method that only allows biotinylation of retrotranslocated molecules by cytosolic BirA protein, recent experiments indicate that both CD4 and tetherin remain glycosylated and retain disulfide bonds during retrotranslocation (Petris et al., 2014). These data suggest that the typical Sec61 channel used for translocation is insufficiently wide to accommodate retrotranslocation substrates modified with these structures (Petris et al., 2014), but an alternative model involving lipid droplet formation has not been confirmed (Olzmann and Kopito, 2011). Given the large number of proteins that have been implicated, a single mechanism for retrotranslocation is unlikely. Despite common delivery of substrates to the proteasome via the p97 ATPase, each of the previous examples of viral ERAD targeting involves different E3 ligases.

Recent evidence suggests that ERAD can target the retrovirus HIV-1 Env (Zhou et al., 2014), a glycosylated transmembrane protein. Studies of a human CD4+ T-cell line CEM.NKR indicated that HIV-1 replication is restricted in these cells, which also are resistant to natural killer cell-mediated lysis (Howell et al., 1985). Surprisingly, these cells overexpressed a mitochondrial translocator protein, TSPO (Braestrup and Squires, 1977; Papadopoulos et al., 2006), and knockdown or knockout of this protein rescued Env and HIV-1 production (Zhou et al., 2014). Further experiments indicated that drugs inducing ERAD led to recovery of Env levels and viral titers. These results suggested that the ER and mitochondria communicate through juxtaposition of their membranes, so that conditions in the mitochondria influence protein folding and ERAD. In support of this conclusion, gp78 is an ERAD-associated E3 ligase (Fang et al., 2001) localized to mitochondria–ER membrane contacts (Fu et al., 2013). Thus, mitochondria proteins may influence ERAD and modulate HIV-1 Env presentation to the immune system.

Triggering of an innate immune response to viruses is affected by the ERAD process. Some anti-viral signaling is controlled through mitochondria, which also cooperates with the ER for lipid synthesis and calcium-controlled processes at the mitochondrial-associated membrane (MAM; Jacobs et al., 2014). Mitochondrial antiviral signaling protein (MAVS; also called IPS-1, VISA, or CARDIF) binds to different retinoic acid-inducible gene-I (RIG-I)-like receptor (RLR) proteins, which sense cytosolic viral RNAs (Kawai et al., 2005; Meylan et al., 2005; Seth et al., 2005; Xu et al., 2005). The MAVS protein is present in the mitochondrial and peroxisomal membranes, and viral RNA triggers both interferon-dependent or independent responses, respectively, (Jacobs and Coyne, 2013; Jacobs et al., 2014). The levels of MAVS are affected by gp78, an E3 ubiquitin ligase that is localized to the ER-mitochondrial interface (MAM; Jacobs et al., 2014). The gp78 ligase was detected by a high throughput RNAi screen to identify genes that restricted enterovirus replication (Coyne et al., 2011). Downregulation of gp78 was shown to decrease yields of vesicular stomatitis virus (VSV) and to increase type I interferon responses.

Some viruses, such as those inducing hepatitis B (HBV) or C (HCV), use ERAD to reduce the amounts of glycoproteins and particles produced. Interestingly, both viruses partially induce the unfolded protein response (UPR; Li et al., 2007, 2011; Saeed et al., 2011), which then increases the levels of certain ERAD components. HBV, a member of the *Hepadnaviridae*, triggers upregulation of the glycoside hydrolase 47 family enzymes, EDEM 1 and 2. Increased EDEM levels appear to bypass normal ER folding of HBV glycoproteins to result in ERAD (Lazar et al., 2012). HCV, a member of the *Flaviviridae*, induces primarily EDEM1 through the UPR and splicing of X-box binding protein 1. Further experiments suggested that elevated levels of EDEM 1 and 3 increase binding to SEL1L, an adapter to the retrotranslocon (**Figure 1**). Inhibition of EDEM binding to SEL1L interfered with ubiquitination of HCV Env protein, E2 (Saeed et al., 2011). Interestingly, infections by another member of the *Flaviviridae*, Japanese encephalitis virus, did not result in EDEM binding to the Env proteins, indicating that not all viral family members control Env proteins by this mechanism. Overall, manipulation of EDEM levels appears to be a common mechanism to reduce viral glycoprotein levels. Lowered amounts of Env proteins and virus particles then contribute to avoidance of innate and adaptive immunity, leading to chronic infections (Saeed et al., 2011; Lazar et al., 2012).

## VIRAL ESCAPE FROM ERAD

A number of pathogens harness the ERAD process to facilitate various replication strategies. The best known examples are the bacterial AB toxins, particularly cholera toxin, which is thought to hijack the ERAD machinery for delivery to the cytosol (Hazes and Read, 1997). Cholera toxin has a catalytic A chain divided into two subunits (CTA1 and CTA2) inside a pore composed of five receptor-binding B subunits (Spangler, 1992). The holotoxin binds to the ganglioside GM1 on the surface of gut epithelial cells, which then triggers toxin internalization and trafficking through the Golgi to the ER (Fujinaga et al., 2003). The A subunits are bound to the B subunits by disulfide bonds, and the toxin complex interacts with the ER-resident enzyme PDI (**Figure 1**). PDI is a redox-dependent chaperone that unfolds the toxin, which is then released in the oxidized state (Tsai et al., 2001). This unfolding event appears to be required for the ability of CTA1 to retrotranslocate to the cytosol, where it induces the ADP-ribosylation of the Gαs protein and, ultimately, opening of chloride channels leading to massive diarrhea (Muanprasat and Chatsudthipong, 2013).

As noted above, retrotranslocation of ERAD substrates is preceded by a recognition step. The chaperone BiP, which is known to be involved in identification of non-glycosylated ERAD substrates, and an ER-resident ATPase (Torsin A) promote CTA1 retrotranslocation (Tsai et al., 2001; Winkeler et al., 2003; Forster et al., 2006; Moore et al., 2010). Sel1L and ERdj5, a co-chaperone of BiP, also facilitate CTA1 retrotranslocation, where the J domain of ERdj5 is required (Williams et al., 2013). ERdj5 also binds to Sel1L, likely providing interaction with the Hrd1 E3 ligase (see **Figure 1**). Torsin A may provide the link to the membrane-resident Derlin-1 protein (Nery et al., 2011). CTA1 retrotranslocation appears to involve Derlin-1 (Bernardi et al., 2008) and the transmembrane ubiquitin ligases, Hrd1 and gp78 (Bernardi et al., 2010). Thus, multiple low affinity interactions are likely involved in the identification of CTA1 as a substrate and its delivery to the retrotranslocon.

Similar to other retrotranslocated substrates, the cytosolic p97 ATPase participates in CTA1 extraction from the ER membrane (Abujarour et al., 2005; Kothe et al., 2005). Nevertheless, CTA1 subverts the normal ERAD process by avoiding polyubiquitination (Rodighiero et al., 2002). The hypothesis that CTA1 avoids ubiquitination through the absence of lysines targeted for polyubiquitination was not substantiated by mutational analysis (Rodighiero et al., 2002). These results indicate that CTA1 employs many of the typical components used for ERAD targeting, including the E3 ligase, but it is unclear how polyubiquitination and degradation of the substrate are avoided. Therefore, retrotranslocon targeting and substrate extraction from the ER membrane is not necessarily coupled to ubiquitination, although ubiquitination may be required for proteasomal degradation.

Viral pathogens also use ERAD. Mouse mammary tumor virus (MMTV) is a betaretrovirus that subverts the ERAD process to complete its viral replication cycle. All retroviruses synthesize an unspliced viral RNA that requires export from the nucleus to the cytosol for translation or packaging into virus particles (Cullen, 2003). The unspliced RNAs of simple retroviruses have a highly structured *cis*-acting sequence, such as the constitutive transport element (CTE) of Mason-Pfizer monkey virus (MPMV; Bray et al., 1994). The CTE facilitates RNA export through the typical TAP/NXF1-mediated pathway used by cellular mRNAs (Grüter et al., 1998). In contrast, the complex retroviruses encode an adapter protein, such as the Rev protein of HIV-1 (Hanly et al., 1989), which binds to a structured RNA element near the 3′ end of the genome (Daly et al., 1989; Zapp and Green, 1989). MMTV also produces a Rev-like protein, Rem, for export of unspliced RNA (Mertz et al., 2005), but Rem binding to viral RNA has additional translation-associated functions (Mertz et al., 2009b).

Unlike other complex retroviruses, Rem is made from an internally deleted form of the Env protein, and the export function resides in a long SP of 98 amino acids (Indik et al., 2005; Mertz et al., 2005). Interestingly, Rem is a precursor protein that is directed to the ER membrane for translation, where it appears to be cleaved by signal peptidase into the Rev-like Rem-SP and a C-terminal glycosylated product (Rem-CT) of unknown activity (Byun et al., 2010). Recent evidence indicates that Rem-SP uses retrotranslocation for extraction from the ER membrane, but, like cholera toxin, avoids proteasomal degradation (Byun et al., 2010, 2012).

**FIGURE 4:**
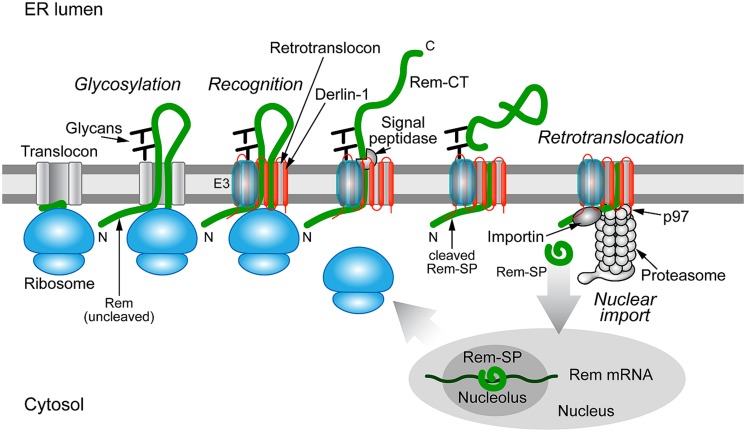
**Trafficking of the MMTV Rem protein by subversion of ERAD.** Rem is a precursor protein that has an N-terminal signal peptide (Rem-SP) that directs translation to the ER membrane. The Rem-CT enters the ER lumen, where it is modified by N-glycosylation on two different sites. Rem recognition for retrotranslocation is not understood, but appears to involve Derlin-1 and, potentially, an E3 ligase, although ubiquitinated Rem has not been observed. Full-length Rem is cleaved by signal peptidase, and Rem-CT is released into the ER lumen. Similar to other retrotranslocation substrates, Rem-SP is extracted from the ER membrane using the p97 ATPase. Despite its dislocation into the cytosol, Rem-SP escapes the proteasome and translocates into the nucleus for binding of MMTV RNA. This figure is adapted from Byun et al. (2012).

Dultz et al. (2008) first reported that Rem is directed to the ER membrane for translation and cleavage by signal peptidase. They also suggested that the Rem precursor (the uncleaved protein) could be detected in the nucleus by fluorescence microscopy (Dultz et al., 2008). Byun et al. (2010) showed that mutation of the predicted signal peptidase cleavage site prevented the appearance of Rem-SP as detected by both Western blotting and a highly sensitive reporter assay for Rev-like function (Mertz et al., 2005; Byun et al., 2010). This assay requires binding to a specific RNA element in viral RNA (Müllner et al., 2008; Mertz et al., 2009a). Fluorescence experiments indicated that only the cleaved Rem-SP enters the nucleus, whereas the uncleaved form was highly unstable and localized to the cytosol (Byun et al., 2010). Furthermore, Rem-SP activity was inhibited by expression of a dominant-negative form of the p97 ATPase required for retrotranslocation (Byun et al., 2010). Rem-SP function also was reduced by the expression of a dominant-negative Derlin-1, but not Derlin-2 protein (Byun et al., in preparation). These results strongly suggest that Rem must be cleaved by signal peptidase prior to SP retrotranslocation to the cytosol and import into the nucleus for RNA binding (**Figure 4**).

Experiments indicate that an altered conformation of either the N-terminal Rem-SP in the cytosol or the ER-luminal portion of Rem affect folding and accessibility to signal peptidase, which is associated with translocons (Falk and Gilula, 1998). First, Rem tagging at the C-terminus with green fluorescent protein (Rem-GFP) resulted in a stable protein that was inefficiently cleaved and had little fluorescence (Mertz et al., 2005; Byun et al., 2012). Rem-GFP also had very low functional activity in reporter assays (Mertz et al., 2005). In contrast, Rem tagged at the N-terminus with GFP was cleaved normally, and GFP-Rem-SP localized to the nucleoli, a result typical of other Rev-like proteins (Cullen, 2003; Mertz et al., 2005). Second, deletion mutations of the Rem C-terminus greatly affected stability of the protein (Byun et al., 2012). Removal of the 50 C-terminal amino acids had little effect on the cleavage or stability of the protein, but deletion of 100 or 150 amino acids produced a highly unstable precursor that could be rescued by the proteasomal inhibitor MG-132 (Byun et al., 2012). Reduced cleavage of the precursor also was observed. Surprisingly, further deletion to give only the SP (Rem-SP) again yielded a stable protein (Byun et al., 2012). Third, substitution of the leucine at position 71 in the SP gave a stable precursor protein that was poorly cleaved by signal peptidase (Mertz et al., 2009a; Byun et al., 2010). An independent report indicated that residues 80 through 98 act as the hydrophobic membrane anchor sequence, suggesting that position 71 is localized in the cytosol (Dultz et al., 2008). Recognition of Rem C-terminal sequences in the ER lumen, presumably by their interaction or lack of interaction with specific chaperone proteins, prevent degradation by ERAD.

The ER-luminal chaperone BiP has repeatedly been detected after purification and proteomic analysis of Rem-binding proteins (Gou et al., manuscript in preparation). Our preliminary data indicate that Rem-SP is not ubiquitinated, and it is possible that this feature protects Rem-SP from proteasomal degradation. Since the Rem precursor and C-terminal deletion mutants are subject to ERAD, cleavage and association with specific cellular proteins appear to be critical for avoidance of the degradative process. The idea that viral proteins manipulate E3 enzymes to form alternative complexes (Olzmann et al., 2013) would be consistent with Rem-SP escape from ERAD.

**FIGURE 5:**
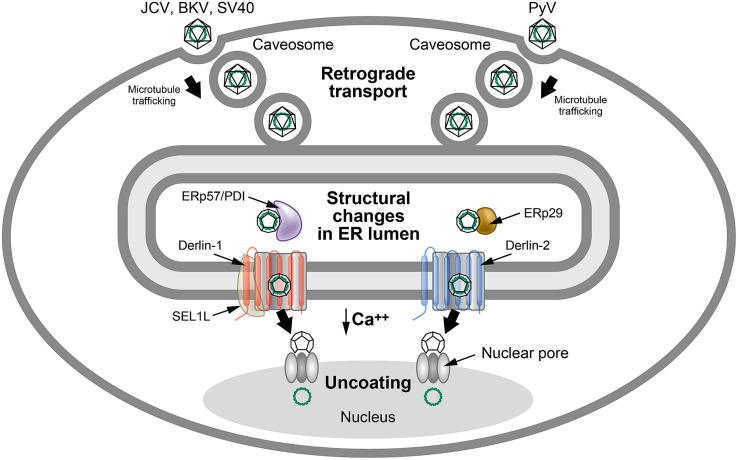
**Use of ERAD for polyomavirus uncoating.** Many polyomaviruses enter through caveosomes that are enriched for viral entry receptors, triggering particle uptake through endosomes. Using the microtubule network, vesicles traffic the virus to the ER, where the unique environment allows structural changes to the icosahedral capsids. Studies of JCV, BKV, and SV40 indicate that viral particles interact with PDI and ERp57 in the ER lumen to rearrange capsid proteins. In contrast, the related mouse polyomavirus (PyV) uses the PDI family member, ERp29, presumably for a similar function. The altered particles then appear to engage different retrotranslocons (dependent on either Derlin-1 or Derlin-2) to induce retrotranslocation to the cytosol, where the reduced calcium environment produces further capsid rearrangements. These particles then bind to the nuclear pore where uncoating occurs to allow passage of viral DNA into the nucleus. This figure is adapted from Neu et al. (2009).

The polyomaviruses have a unique entry method that uses retrotranslocation, while avoiding ERAD. The BK polyomavirus (BKV) first binds to the ganglioside receptors GT1b and GD1b and enters through caveolae (Neu et al., 2009), which are composed of membrane microdomains/lipid rafts that are enriched for sphingolipids and signaling molecules (Head et al., 2014; **Figure 5**). Particle delivery to the cytosol occurs through a pH-dependent step involving endosomal trafficking via microtubules to the ER (Eash and Atwood, 2005; Moriyama and Sorokin, 2008; Jiang et al., 2009). Other members of the Polyomaviridae use caveolae-independent entry for ER delivery (Neu et al., 2009). ER localization of these viruses is necessary to access specific retrotranslocation components.

The VP1 capsid proteins of polyomaviruses form pentamers during assembly that are held together by disulfide bonding (Li et al., 2003). Each pentamer is associated with one molecule of either the minor capsid protein VP2 or VP3 (Barouch and Harrison, 1994), which become accessible to antibodies after exposure to the unique environment of the ER (Norkin et al., 2002). Particle delivery into the ER allows reduction and isomerization of disulfide bonds using ERp29 (mouse polyomavirus; Magnuson et al., 2005) or ERp57 and PDI (SV40; Schelhaas et al., 2007) to allow partial uncoating (Jiang et al., 2009; Tsai and Qian, 2010). The partially uncoated virion then engages the retrotranslocation machinery to allow cytosolic entry similar to cholera toxin (Neu et al., 2009).

Interestingly, different polyomaviruses use distinct Derlin family members for retrotranslocation. SV40 uses Derlin-1 and SEL1L (Schelhaas et al., 2007), whereas mouse polyoma virus uses Derlin-2 (Lilley et al., 2006; **Figure 5**). Additional experiments indicate that exposure of VP2 hydrophobic sequences tethers virus particles to the ER membrane, and that both BiP and BAP31 are needed for dislocation of SV40 to the cytosol (Geiger et al., 2011). BAP31 may serve as a shuttle to the ERQC that has been associated with enriched ERAD components (Kamhi-Nesher et al., 2001; Wakana et al., 2008). Furthermore, use of epoxomicin or eeyarestatin 1, inhibitors of the proteasome or p97 ATPase, respectively, blocked early events of BKV infection (Bennett et al., 2013). Epoxomicin treatment of cells allowed accumulation of BKV in the calnexin-rich, BiP-deficient ERQC (Bennett et al., 2013). These results are consistent with ERAD extraction of polyomaviruses from the ER to the cytosol, although it is has been suggested that there are cell-type and virus-specific differences and that direct ER to nuclear transport may occur (Bennett et al., 2013). Low levels of calcium in the cytosol lead to further capsid destabilization and exposure of the nuclear localization signals on capsid proteins. The partially uncoated capsid then transits through the nuclear pores for initiation of viral DNA replication (Neu et al., 2009).

The preceding experiments indicate that ERAD is used by viruses to allow trafficking events that promote replication. MMTV Rem trafficking through the ER allows access to signal peptidase and cleavage of Rem precursor into functional N- and C-terminal proteins. In contrast, the polyomaviruses use ERAD to partially uncoat virions on their path to the nucleus. Importantly, both types of viruses avoid proteasomal degradation during ERAD, although the mechanisms remain unclear.

## VIRUSES AND ERAD TUNING

ERAD may be regulated or “tuned” through the rapid turnover of specific components through the proteasomes or autophagosomes/vesicular trafficking to lysosomes (Merulla et al., 2013). Normal secretory vesicles released from the ER are 60–70 nm in diameter and have coatamer proteins, such as COPII, whereas the ER-derived tuning vesicles (EDEMosomes) lack coatamers and are 200–800 nm in diameter (Bernasconi et al., 2012b). Tuning vesicles contain SEL1L, EDEM1, and OS-9, which are transmembrane or luminal proteins involved in ERAD (**Figure 1**; Olzmann et al., 2013). EDEMosomes are believed to reduce ERAD by disposal in acidic organelles (Bernasconi et al., 2012b), favoring the correct folding of polypeptides (Calì et al., 2008). The coronaviruses are known to take advantage of ERAD tuning (Reggiori et al., 2010).

Many plus-stranded RNA-containing viruses manipulate cellular membranes to further RNA replication (Paul and Bartenschlager, 2013). These membrane structures have been divided into invaginated vesicle/spherule type and double-membrane vesicle (DMV) type (two lipid bilayers). Such vesicles allow viruses to concentrate their replication components, to separate distinct viral processes (e.g., translation, transcription, and replication), and to avoid immune detection (Paul and Bartenschlager, 2013). Severe acute respiratory syndrome coronavirus (SARS-CoV) and mouse hepatitis virus (MHV) induce DMVs for targeting their replication and transcription (Reggiori et al., 2010). The DMVs originate from ER membranes and contain the non-structural transmembrane proteins nsp3 and nsp4 and viral double-stranded RNA (Stertz et al., 2007; Reggiori et al., 2010). Nevertheless, DMVs lack markers typical of the ERGIC or the Golgi (Oostra et al., 2007).

Recent experiments indicate that DMVs are coated with microtubule-associated protein light chain 3 [LC3; Atg8 in yeast (Reggiori et al., 2010)], which is a ubiquitin-like modifier (van der Veen and Ploegh, 2012). LC3 can exist in a lipidated form (covalent linkage to phosphatidylethanolamine; also known as LC3-II) or a predominantly cytosolic non-lipidated form (LC3-I). LC3-II is believed to be involved in fusion of autophagosomes to lysosomes (van der Veen and Ploegh, 2012), but coronavirus DMVs display the non-lipidated LC3-I (Reggiori et al., 2010). These ubiquitin-like modifiers recognize specific receptors that target associated vesicles to particular cellular locations (van der Veen and Ploegh, 2012). The coronaviruses appear to be redirecting vesicles destined for autophagosomes to sequestered locations in the cytosol where replication will occur.

The autophagy machinery is not required for coronavirus replication, and no colocalization of viral non-structural proteins was observed with LC3-II-coated autophagosomes (Reggiori et al., 2010). Coronavirus-induced DMVs and EDEMosomes both are coated with the non-lipidated LC3-I protein (Calì et al., 2008; Reggiori et al., 2010), which is not covalently attached to membranes like LC3-II (Kabeya et al., 2000). Induction of autophagy in coronavirus-infected cells with rapamycin decreased the levels of EDEM1 and coronavirus (Reggiori et al., 2010). The virus-containing DMVs had both EDEM1 and OS-9, but not other ERAD-associated chaperones, and virus infection interfered with ERAD tuning by hijacking the EDEMosomes. Nevertheless, LC3-I, but not EDEM1 and OS-9, is necessary for coronavirus infection, and the hijacked EDEMosome cargo is not degraded by proteases in the endosomes/lysosomes (Reggiori et al., 2010). Further, the ERAD transmembrane adapter protein, SEL1L, is needed for DMV formation, capturing the ER-resident EDEM1 and OS-9 proteins (and possibly XTP3-B and EDEM3), while using its proline-rich cytosolic domain to bind to LC3-I. As expected, SEL1L knockdown impairs coronavirus replication (Bernasconi et al., 2012a).

The organizationally similar arterioviruses (classified with coronaviruses, toroviruses, and roniviruses into the order Nidovirales; Gorbalenya et al., 2006) subvert EDEMosome trafficking for their replication, although the size of the vesicles is smaller (Monastyrska et al., 2013). The mechanism for altering EDEM1-containing vesicular trafficking is unclear, but likely involves expression of viral non-structural proteins that span the ER-derived membranes (Monastyrska et al., 2013), perhaps through their interaction with SEL1L. These experiments indicate that viruses hijack EDEMosomes to sequester their double-stranded RNA from cytosolic sensors that will trigger interferon production and innate immunity (Zinzula and Tramontano, 2013). Other components of the ERAD system, particularly chaperone proteins, also participate in the replication and transmission of both plant and mammalian viruses (Verchot, 2014).

## CONCLUSION

The ERAD system is a complex and highly regulated process controlling the disposal of misfolded or misassembled proteins that are directed to the ER for translation. Deregulation of this process results in pathogenic conditions, including infectious diseases. Viruses exploit ERAD to decrease overall viral levels and allow establishment of chronic infections by minimizing antigen presentation to the immune system. Trafficking of specific viral proteins or entire virion particles may involve ERAD for refolding or processing in the unique ER environment. Alternatively, viruses can use ERAD-associated components to form isolated lipid vesicles for replication and shelter from immune detection. Virus-mediated subversion of ERAD can lead to degradation of molecules that are involved in innate or adaptive immunity. Continued studies of viruses are certain to provide additional insights into both the ERAD process and the components that regulate it. Further experiments may identify targets for viral therapeutics.

## Conflict of Interest Statement

The authors declare that the research was conducted in the absence of any commercial or financial relationships that could be construed as a potential conflict of interest.
